# Epilepsy and seizures in young people with 22q11.2 deletion syndrome: Prevalence and links with other neurodevelopmental disorders

**DOI:** 10.1111/epi.14722

**Published:** 2019-04-11

**Authors:** Christopher B. Eaton, Rhys H. Thomas, Khalid Hamandi, Gareth C. Payne, Michael P. Kerr, David E. J. Linden, Michael J. Owen, Adam C. Cunningham, Ullrich Bartsch, Siske S. Struik, Marianne B. M. van den Bree

**Affiliations:** ^1^ Medical Research Council Centre for Neuropsychiatric Genetics and Genomics Division of Psychological Medicine and Clinical Neurosciences Cardiff University School of Medicine Cardiff UK; ^2^ Cerebra Centre for Neurodevelopmental Disorders School of Psychology University of Birmingham Birmingham UK; ^3^ Institute of Neuroscience Newcastle University Newcastle‐upon‐Tyne UK; ^4^ The Epilepsy Unit University Hospital of Wales Cardiff UK; ^5^ Betsi Cadwaladr University Health Board Wales UK; ^6^ School for Mental Health and Neuroscience Faculty of Health Medicine and Life Sciences Maastricht University Maastricht The Netherlands; ^7^ School of Physiology, Pharmacology and Neuroscience University of Bristol Bristol UK; ^8^ Lilly UK Erl Wood Manor Surrey UK; ^9^ Immunodeficiency Centre for Wales University Hospital of Wales Cardiff UK

**Keywords:** electroencephalography, febrile seizure, psychiatric disorder, seizure semiology, unprovoked seizure

## Abstract

**Objective:**

The true prevalence of epileptic seizures and epilepsy in 22q11.2 deletion syndrome (22q11.2DS) is unknown, because previous studies have relied on historical medical record review. Associations of epilepsy with other neurodevelopmental manifestations (eg, specific psychiatric diagnoses) remain unexplored.

**Methods:**

The primary caregivers of 108 deletion carriers (mean age 13.6 years) and 60 control siblings (mean age 13.1 years) completed a validated epilepsy screening questionnaire. A subsample (n = 44) underwent a second assessment with interview, prolonged electroencephalography (EEG), and medical record and epileptologist review. Intelligence quotient (IQ), psychopathology, and other neurodevelopmental problems were examined using neurocognitive assessment and questionnaire/interview.

**Results:**

Eleven percent (12/108) of deletion carriers had an epilepsy diagnosis (controls 0%, *P* = 0.004). Fifty‐seven of the remaining 96 deletion carriers (59.4%) had seizures or seizurelike symptoms (controls 13.3%, 8/60, *P* < 0.001). A febrile seizure was reported for 24.1% (26/107) of cases (controls 0%, *P* < 0.001). One deletion carrier with a clinical history of epilepsy was diagnosed with an additional type of unprovoked seizure during the second assessment. One deletion carrier was newly diagnosed with epilepsy, and two more with possible nonmotor absence seizures. A positive screen on the epilepsy questionnaire was more likely in deletion carriers with lower performance IQ (odds ratio [OR] 0.96, *P* = 0.018), attention‐deficit/hyperactivity disorder (ADHD) (OR 3.28, *P* = 0.021), autism symptoms (OR 3.86, *P* = 0.004), and indicative motor coordination disorder (OR 4.56, *P* = 0.021).

**Significance:**

Even when accounting for deletion carriers diagnosed with epilepsy, reports of seizures and seizurelike symptoms are common. These may be “true” epileptic seizures in some cases, which are not recognized during routine clinical care. Febrile seizures were far more common in deletion carriers compared to known population risk. A propensity for seizures in 22q11.2DS was associated with cognitive impairment, psychopathology, and motor coordination problems. Future research is required to determine whether this reflects common neurobiologic risk pathways or is a consequence of recurrent seizures.

**Key points**

- Over half of young people with 22q11.2 deletion syndrome (22q11.2DS) without an epilepsy diagnosis were reported as having a seizure or seizure‐like symptom
- We conducted a second assessment stage in a subsample of 44 people including an interview, a prolonged electroencephalography (EEG) assessment, and a medical record review
- This highlighted that in some deletion carriers, epileptic seizures may not be recognized during routine clinical care and that epilepsy may be overlooked
- A quarter of young people with 22q11.2DS screened positive for febrile seizures, a higher rate than reported in previous studies in this population
- Epilepsy, seizures, and seizurelike symptoms were associated with attention‐deficit/hyperactivity disorder (ADHD), autism spectrum disorder (ASD) symptoms, motor coordination problems, and a lower performance intelligence quotient (IQ) in 22q11.2DS

## 1 INTRODUCTION

The most common recurrent microdeletion syndrome in humans is 22q11.2 deletion syndrome (22q11.2DS), which occurs in ~1 in 2000‐4000 live births.^1^ In the majority of cases, a de novo 3 megabase (Mb) deletion occurs between low copy repeats (LCRs) 22A‐22D, causing the loss of 46 protein‐coding genes.^2^ This deletion syndrome has a variable phenotype, with characteristic features including congenital heart disease, palatal abnormalities, hypocalcemia, mild‐moderate intellectual disability (ID), and psychiatric disorders such as schizophrenia, attention‐deficit/hyperactivity disorder (ADHD), anxiety disorder and autism spectrum disorder (ASD).^3^, ^4^, ^5^, ^6^, ^7^, ^8^, ^9^, ^10^, ^11^

The deletion increases the risk of acute symptomatic and unprovoked epileptic seizures, although the reported rates are wide‐ranging. Between 1% and 14.5% of patients have hypocalcemia‐induced seizures.^12^, ^13^, ^14^, ^15^, ^16^ In adulthood, 17.6% of patients with 22q11.2DS exposed to psychotropic drugs have epileptic seizures, suggesting a reduced seizure threshold.^13^ Between 4.4% and 36.8% have repeated unprovoked seizures (ie, epilepsy).^9^, ^13^, ^14^, ^15^, ^17^, ^18^ Structural brain abnormalities in these individuals can include diffuse cerebral atrophy (18.8%), polymicrogyria (13.9%), hippocampal malrotation (10.9%), gray and white matter heterotopia (5.9%), and focal cortical dysplasia (2%).^19^ Between 1% and 6.9% of patients have genetic generalized epilepsy (GGE),^13^, ^14^, ^15^, ^17^ and the deletion prevalence is elevated in GGE cohorts.^20^

To date, studies investigating the prevalence of epileptic seizures and epilepsy in deletion carriers have relied on retrospective medical record review. Such records are less suited to systematic evaluation, because of differences between clinicians in diagnostic methods and documentation. This approach may furthermore miss cases of nonconvulsive seizures (eg, absences) not seen clinically. Nonconvulsive seizures normally occur several times before the affected individual and their family seek clinical advice.^21^ Andrade et al (2013) were the first to conduct a direct, systematic study of epilepsy in 22q11.2DS, including epileptologist, magnetic resonance imaging (MRI), and prolonged electroencephalography (EEG) assessment. They observed that 36.8% of patients had epilepsy, although their sample consisted of 19 adults only.^18^ Thus, previous research may have underestimated the prevalence of epileptic seizures and epilepsy in 22q11.2DS, particularly in young people. In addition, there is a dearth of research into salient seizure characteristics in 22q11.2DS, such as length and frequency, increases in which associate with poorer neurodevelopmental outcomes in the general population.^22^, ^23^

Studies based primarily on historical medical record review have also assessed the relationships of epileptic seizures and epilepsy with cognitive and psychiatric development in 22q11.2DS. Neonatal seizures (mostly hypocalcemia‐induced) predict poorer intellectual functioning later in life.^16^ Epilepsy also associates with a higher rate of developmental delay, but not with psychiatric disorder.^17^ The relationships of epilepsy with specific psychiatric diagnoses (eg, ADHD) and other salient manifestations of 22q11.2DS, such as sleep disturbance^24^ and motor coordination problems,^4^ have not been explored.

Our study had three aims: First, we explored the rates of an epilepsy diagnosis, seizures, and seizurelike symptoms (behaviors that may reflect unrecognized epileptic seizures) in young people with 22q11.2DS and their unaffected control siblings, through data obtained from a validated questionnaire completed by the primary caregiver. To support these findings, we conducted a second stage of assessment with a subsample, involving primary caregiver interview, prolonged EEG assessment, and a medical record review. These data were then reviewed by an epileptologist, who made diagnoses of epileptic seizures and epilepsy.

Second, we sought to delineate salient seizure characteristics in deletion carriers who took part in the second assessment, such as etiology, semiology, age at onset, length, and frequency.

Third, we investigated whether epilepsy questionnaire responses were predicted by intellectual functioning (IQ and ID), psychopathology (ADHD, anxiety disorder, and indicative ASD), motor coordination problems, and sleep disturbance in deletion carriers.

## 2 METHODS

### 2.1 Participants

We recruited 108 deletion carriers (57.4% male, mean age 13.6 years, standard deviation [SD] 3.3, range 6.2‐20.5) and 60 unaffected control siblings (50% male, mean age 13.1 years, SD 3.2, range 6.3‐18.9) via UK genetics clinics and charities for chromosomal conditions. The deletion was identified in the clinics (fluorescence in situ hybridization/microarray) and confirmed in the MRC Centre for Neuropsychiatric Genetics and Genomics laboratory (microarray). For 15.8% of cases, information about deletion size was not provided and we could not obtain a biologic sample for verification. In the remainder, the majority had the typical de novo ~3 megabase (Mb) A‐D deletion and all were haploinsufficient for *TBX1*, except for one atypical B‐D deletion (Table 1). We obtained informed written consent from primary caregivers and participants. The National Health Service Wales Research Ethics Committee approved our protocols.

**Table 1:** Descriptive statistics of the sample

	%
Deletion etiology	
De novo	77.8
Inherited	8.3
Unknown	13.9
Deletion type	
~3 Mb A‐D	75.9
~2 Mb A‐C	0.9
~1.5 Mb A‐B	6.5
Atypical ~1.5 Mb B‐D	0.9
Unknown	15.8
Family ethnic background	
European	85.2
Mixed	10.2
Non‐European	1.9
Unknown	2.8
Highest parental qualification^a^	
Low (O‐levels/GCSEs)	23.2
Middle (A‐levels/highers/vocational training)	32.4
High (university degree and/or other higher postgraduate qualification)	32.4
Unknown	12.0
Family income	
≤£19 999	22.2
£20 000‐£39 999	24.1
£40 000‐£59 999	21.3
≥£60 000	23.1
Unknown	9.3

	Mean	SD	Range
Age			
22q11.2DS	13.6	3.3	6.2‐20.5
Controls	13.1	3.2	6.3‐18.9

	Male (%)	Female (%)
Gender		
22q11.2DS	62 (57.4)	46 (42.6)
Controls	30 (50)	30 (50)

GCSE, General Certificate of Secondary Education; 22q11.2DS, 22q11.2 deletion syndrome.

a Of the parent completing the questionnaire.

### 2.2 Screening for lifetime history of epilepsy, seizures, and seizurelike symptoms

We used the validated Epilepsy Screening Questionnaire (ESQ; Ottman et al,^25^ Table 2) to screen for an epilepsy diagnosis (Item 9), seizures (Items 1 and 2) and seizurelike symptoms (behaviors that may reflect unrecognized epileptic seizures, Items 3‐8). We created two dichotomous summary variables: “any positive,” a positive response (“yes” or “possibly”) to at least one item; and “any positive excluding epilepsy,” a positive response to any of Item 1 to Item 8 in those without epilepsy. The ESQ was originally self‐report^25^; however, we asked the primary caregiver to complete it in all cases, given the age and presence of mild‐moderate ID in our 22q11.2DS sample.^3^ In the Ottman et al^25^ validation study, participants were only asked about afebrile seizures and seizurelike symptoms if they didn't have epilepsy. We asked all items, to probe for unrecognized seizures in epilepsy patients.

**Table 2:** Rates of positive responses on each item of the Epilepsy Screening Questionnaire in young people with 22q11.2DS and their unaffected control siblings

Item	Positive response		χ^2^	OR	*P* value
	22q11.2DS, n (%)	Controls, n (%)			
1. Did your son/daughter ever have a seizure or convulsion caused by a high fever?^a^	26 (24.3)	0 (0)	17	_	<0.001
2. Other than the seizures associated with high fevers, has your son/daughter ever had a seizure, convulsion, fit, or spell—under any circumstances?^b^	26 (24.1)	0 (0)	16.8	_	<0.001
3. Other than the seizures associated with high fevers, has your son/daughter ever had uncontrolled movements of part or all of his/her body such as twitching, jerking, shaking, or going limp?^c^	21 (19.8)	2 (3.3)	8.72	7.1	0.003
4. Other than the seizures associated with high fevers, has your son/daughter ever had an unexplained change in his/her mental state or level of awareness; or an episode of “spacing out” that he/she could not control?^d^	19 (17.8)	1 (1.7)	9.44	12.6	0.002
5. Does your son/daughter daydream or stare into space more than other children?	43 (40.2)	5 (8.3)	19	7.31	<0.001
6. Have you ever noticed him/her to have any unusual body movements when exposed to strobe lights, video games, flickering lights, or sun glare?	3 (2.8)	0 (0)	Fisher's exact test	_	0.553
7. Shortly after waking up, either in the morning or after a nap, have you ever noticed your son/daughter have uncontrollable jerking or clumsiness, such as dropping things or things suddenly flying from his/her hands?	10 (9.3)	0 (0)	Fisher's exact test	_	0.014
8. Has your son/daughter ever had any other type of repeated unusual spells?	7 (6.5)	0 (0)	Fisher's exact test	_	0.051
9. Has your son/daughter ever been diagnosed with a seizure disorder or epilepsy?	12 (11.1)	0 (0)	Fisher's exact test	_	0.004

22q11.2DS, 22q11.2 deletion syndrome; OR, odds ratio.

a Data available for 107 probands and 59 control siblings.

b Data available for 59 control siblings.

c Data available for 106 probands.

d Data available for 107 probands.

### 2.3 Second stage of assessment of epileptic seizures and epilepsy

To validate our screening findings, we conducted a second assessment in a subsample of 40 deletion carriers and 4 controls with “any positive.”

A trained researcher interviewed the primary caregiver about ESQ‐reported events (for 37 deletion carriers and 4 controls), using an amended version of the validated Seizure Classification Interview (SCI), developed by Ottman et al.^26^, ^27^ Diagnoses from the SCI show fair to excellent nonchance agreement with neurologist diagnoses in studies of epilepsy patients (*k* = 0.54‐0.83).^26^ We used the interview to establish whether ESQ‐reported events were epileptic seizures. We therefore combined the “grand mal” and “small seizures” sections into an “unusual spell” section. In addition, we added a section on febrile seizures specifically. We undertook medical record reviews where available (11 deletion carriers).

We conducted prolonged ambulatory EEG with 28 deletion carriers and 4 controls, with overnight video‐monitoring (22q11.2DS: mean recording length 13:41:16, SD 04:59:40, range 3:30:00‐21:03:06, controls: mean 11:14:38, SD 05:14:36, range 03:25:00‐14:25:23). Recordings were performed using a 64‐channel Hydrocel Geodesic Sensor Net (Electrical Geodesics Inc.) and a BE PLUS LTM amplifier (EB Neuro S.p.A.).

A consultant epileptologist and a consultant neurophysiologist reviewed the EEG traces, and participants had their epileptic seizures classified and epilepsy diagnosed by the epileptologist, according to International League Against Epilepsy (ILAE) criteria.^28^, ^29^, ^30^

### 2.4 IQ and psychopathology

We derived the child's full‐scale IQ (FSIQ), performance IQ (PIQ) and verbal IQ (VIQ) scores from the Wechsler Abbreviated Scale of Intelligence (WASI).^31^ ID was defined as FSIQ <70. We conducted the Child and Adolescent Psychiatric Assessment interview (CAPA)^32^ with the primary caregiver to diagnose ADHD and anxiety disorder, according to Diagnostic and Statistical Manual of Mental Disorders, Fourth Edition, Text Revision (DSM‐IV‐TR)^33^ criteria. We screened for indicative ASD using the Social Communication Questionnaire,^34^ completed by the primary caregiver.

### 2.5 Other neurodevelopmental problems

We screened for indicative developmental coordination disorder (DCD) using the Developmental Coordination Disorder Questionnaire (DCDQ),^35^, ^36^ completed by the primary caregiver. Sleep disturbance was defined as a score of two on one item from the CAPA sleep section. Primary caregivers provided information on preterm birth, cardiovascular problems, recurrent infections, and lifetime medication use.

Sample sizes for the ESQ, IQ, psychopathology, and other neurodevelopmental problem datasets differed due to the child or parent not completing part/all of one or more measures. The primary caregiver was not available for the SCI for three deletion carriers who underwent EEG.

### 2.6 Statistical analyses

Statistical analyses were conducted in R version 3.5.1 (https://www.R-project.org/).

#### 2.6.1 Screening for lifetime history of epilepsy, seizures, and seizurelike symptoms

We used logistic regression analyses to measure associations between deletion status (22q11.2DS/control) and the “any positive” and “any positive excluding epilepsy” variables. Predictors were entered hierarchically: age first, then gender, then deletion status. We conducted a sensitivity analysis to explore whether preterm birth, cardiovascular problems, and lifetime history of psychotropic and/or antiepileptic medication use influenced our findings. We explored associations between deletion status and response to each ESQ item using χ^2^/Fisher's exact test. We conducted a logistic regression exploring whether recurrent infections predicted febrile seizures in deletion carriers.

#### 2.6.2 Second stage of assessment of epileptic seizures and epilepsy

For the “epilepsy diagnosis” and “any positive excluding epilepsy” ESQ variables, we calculated the percentage of deletion carriers who were diagnosed with epilepsy and epileptic seizures by our epileptologist, respectively, as well as the rates of epileptiform discharges in these groups; no background abnormalities were observed. We also calculated the percentage of deletion carriers reported with febrile seizures (Item 1, Table 2) who had their report confirmed by our epileptologist. For controls, we repeated these analyses with the “any positive excluding epilepsy” ESQ variable only (no controls had epileptic seizures or abnormal EEG findings).

We calculated the rate of different seizure etiologies in deletion carriers who were diagnosed with epileptic seizures. We also explored characteristics of febrile and unprovoked seizures (the most common seizure etiologies in our sample) such as the semiology (rates of generalized and focal seizures), median age at onset (months), median number, median length (seconds), and frequency (rates of four different categories; Table 4; eg, less than once a month).

#### 2.6.3 Association of the ESQ with neurodevelopmental problems in 22q11.2DS

In deletion carriers, we used logistic regression analyses to explore the associations of the “any positive” “any positive excluding epilepsy” and “febrile seizure” ESQ variables with IQ scores, ID, psychopathology, indicative DCD, and sleep disturbance. Predictors were entered hierarchically, age first, then gender, and finally the neurodevelopmental variable. We compared FSIQ, VIQ, and PIQ scores and rates of ID, psychopathology, indicative DCD, and sleep disturbance between deletion carriers and controls using *t* tests and χ^2^ tests.

## 3 RESULTS

Table 1 displays descriptive statistics about our sample.

### 3.1 Screening for lifetime history of epilepsy, seizures, and seizurelike symptoms

Table 2 shows the rates of positive responses on each ESQ item. Sixty‐nine of 108 **(**63.9%) deletion carriers had “any positive” compared to 13.3% (8/60) of controls (*b = *2.49, *z *= 5.67, odds ratio [OR] 12, *P* < 0.001). Of deletion carriers, 11.1% (12/108) had an epilepsy diagnosis, yet when excluding these cases 59.4% (57/96) had “any positive excluding epilepsy” compared to 13.3% (8/60) of controls (*b *=* *2.27, *z* = 5.15, OR 9.66, *P* < 0.001). Around a quarter of deletion carriers had febrile seizures (controls 0%, *P* < 0.001, Table 2). Positive responses were significantly more common in deletion carriers than in controls for four of the seizurelike symptom items (Items 3, 4, 5, and 7 in Table 2). Age and gender did not influence these relationships.

Nineteen of 108 (17.6%) deletion carriers had a lifetime history of psychotropic and/or antiepileptic medication use. Fourteen of 103 cases (13.6%) and 4.3% (2/46) of controls were born prematurely; 64.8% (68/105) of cases and 1.8% (1/59) of controls had cardiovascular problems. These comorbidities did not influence our findings.

The majority of deletion carriers had recurrent infections (68.9%, 73/106, controls 10.9%, 6/55; *P* < 0.001). Deletion carriers with recurrent infections were not more likely to report febrile seizures, however (*b *=* *0.78, *z* = 1.41, *P* = 0.157).

### 3.2 Second stage of assessment of epileptic seizures and epilepsy

Deletion carriers in the second assessment did not differ significantly from those who did not take part with respect to their FSIQ score, rate of psychopathology, or highest level of parental education (*P* > 0.05 in all cases), although they were significantly older (14.4 years vs 12.17 years, *P* = 0.005). The results from the second assessment are shown in Table 3. For most deletion carriers reported as having epilepsy, whose caregiver was subsequently interviewed, the diagnosis was confirmed (5/6, 83%). The remaining individual had an isolated unprovoked generalized tonic–clonic seizure (GTCS). One deletion carrier was reported as having epilepsy based on a history of what a pediatrician had described as “petit mal” seizures. We reclassified these as focal nonmotor seizures with impaired awareness, in the context of right‐hemisphere polymicrogyria (reported during the SCI). For this participant, we further diagnosed a report of repeated left‐sided numbness and clumsiness in the morning as focal motor (postictal) seizures, thereby identifying an additional seizure type.

**Table 3:** Results from the second stage of assessment in young people with 22q11.2DS and control siblings

ESQ variable	n screening positive/total n (%)	Interview completed? (%)	Diagnosis confirmed by epileptologist? (%)	EEG completed? (%)	Epileptiform discharges? (%)
22q11.2DS					
ESQ: epilepsy diagnosis	12/108 (11.1)	6/12 (50)	5/6 (83.3)	2/12 (16.7)	0/2 (0)
ESQ: “any positive excluding epilepsy”	57/96 (59.4)	31/57 (54.4)	16/31 (51.6)	26/57 (45.6)	3/26 (11.5)
Controls					
ESQ: “any positive excluding epilepsy”	8/60 (13.3)	4/8 (50)	0/4 (0)	4/8 (50)	0/4 (0)

22q11.2 DS, 22q11.2 deletion syndrome; EEG, electroencephalography; ESQ, Epilepsy Screening Questionnaire.

Just over half of deletion carriers with “any positive excluding epilepsy,” whose caregiver was subsequently interviewed, were diagnosed with epileptic seizures (16/31; 51.6%). In two, the diagnosis was “possible” nonmotor absence seizures (based on a history of daydreaming spells). Their caregivers reported that they had not seen a clinician for these events. Of interest, the EEG for one of these two displayed short bursts of generalized and left hemisphere spike‐and‐slow‐wave discharges, occurring during sleep and shortly after awakening (Figure S1). A further 2 of the 16 individuals also met criteria for epilepsy. The primary caregiver for one described a history of repeated unprovoked GTCS in the SCI, but no epilepsy diagnosis. However, a child development clinic letter listed epilepsy as a diagnosis. For the other individual, we diagnosed a history of repeated “staring spells” as nonmotor absence seizures. Medical records were unavailable, but the primary caregiver reported that a pediatrician had previously described these as “possible absence seizures” and that further assessments had not been conducted as these events had stopped. Of interest, this child showed brief generalized spike‐and‐slow‐wave epileptiform discharges shortly after awakening. The remaining deletion carriers (15/31) were diagnosed with nonepileptic events (eg, daydreaming), although one showed brief generalized, left and right hemisphere spike‐and‐slow‐wave discharges during sleep. Eighty percent of these individuals (12/15) were only reported to have seizurelike symptoms on the ESQ.

Most deletion carriers with ESQ‐reported febrile seizures, whose caregiver was subsequently interviewed, were diagnosed with febrile seizures (16/17, 94%). The remaining individual had fever‐related delirium. All controls reported as having “any positive excluding epilepsy” were diagnosed with nonepileptic events.

Table 4 shows salient characteristics of epileptic seizures in deletion carriers. Febrile and unprovoked epileptic seizures were the most common seizure etiologies (77.3% and 45.5%, respectively). All febrile seizures were convulsive, with a median estimated length of 2.5 minutes. On average, febrile seizures began within the first 2 years of life and were recurrent. Unprovoked seizures were not linked to a structural etiology in 60% of deletion carriers. In the remaining 40%, unprovoked seizures were seen in children with structural brain abnormalities or potential acquired causes; right hemisphere polymicrogyria, perinatal subdural hematoma, possible postpneumococcal meningitis (SCI‐report only), and neonatal hypoxic‐ischemic encephalopathy (SCI‐report and medical records). Unprovoked seizures were mostly generalized, lasted for ~2 minutes on average, and onset within the first 3 years of life. Thirty percent experienced daily unprovoked seizures at their most frequent. These three individuals had focal motor seizures with postictal left‐sided numbness upon awakening; “possible” nonmotor absence seizures; and GTCS with nonmotor absence seizures.

**Table 4:** Rates and characteristics of febrile and unprovoked seizures in young people with 22q11.2DS who took part in the second stage of assessment

Seizure etiology	n/n with epileptic seizures (%)	Seizure semiology (%)		Median age of onset in months (range)^a^	Median seizure length in seconds (range)^b^	Median number of seizures (range)	Frequency (%)^c^			
		Generalised	Focal				Less than once a month	One to four times a month	More than four times a month but less than once a day	Daily or more
Febrile	17/22 (77.3)	17 (100)	0 (0)	14 (0‐168)	150 (10‐900)	2 (1‐7)	–	–	–	–
Unprovoked	10/22 (45.5)	8 (80)	2 (20)	33.0 (0‐108)	135 (3‐330)	–	3 (30)	3 (30)	1 (10)	3 (30)
Genetic	6/22 (27.3)									
Structural	4/22 (18.2)									

a The earliest age when seizures appeared was used for deletion carriers with multiple types of unprovoked seizure. Within the range, “0” refers to deletion carriers who had their first seizure during the first month of life.

b The time of the longest seizure was used for deletion carriers with seizures of differing lengths.

c Primary caregivers were asked to indicate the maximum frequency of seizures for their child.

Other seizure etiologies not shown in Table 4 include hypocalcemia (9.1%, 2/22, GTCS in both cases), cardiac surgery (4.5%, 1/22, focal motor seizure), and hypoxic‐ischemic encephalopathy (4.5%, 1/22, focal motor seizure, possibly to bilateral tonic–clonic).

### 3.3 Association of the ESQ with neurodevelopmental problems in 22q11.2DS

#### 3.3.1 IQ and ID

The mean FSIQ, PIQ, and VIQ of deletion carriers were significantly lower than in controls (Table S1). One deletion carrier had a higher‐than‐average FSIQ (117), whereas 12.1% (12/99) and 40.4% (40/99) were in the average (86‐115) and borderline ranges (71‐85), respectively; 41.4% (41/99) had mild ID (55‐70) and 5.1% had (5/99) moderate ID (IQ < 55). By contrast, 94.5% (52/55) of control siblings had an average‐or‐higher FSIQ and only 3 (5.5%) had a borderline FSIQ. “Any positive” was more common in deletion carriers with a lower PIQ (*P* = 0.018, Table 5). This relationship was not observed for “any positive excluding epilepsy” (*P* = 0.060) or febrile seizures (*P* = 0.105). None of the ESQ variables were predicted by FSIQ, VIQ, or ID. Age and gender did not influence our findings.

**Table 5:** IQ scores and rates of neurodevelopmental problems for young people with 22q11.2DS on the “any positive” summary variable

Measure	n	Any positive		*b*	z	OR	*P* value
		No (SD)	Yes (SD)				
FSIQ	99	73.94 (13.31)	70.35 (11.15)	−0.02	−1.39	0.98	0.163
PIQ	99	78.49 (14.05)	72.31 (10.94)	−0.04	−2.36	0.96	0.018
VIQ	100	73.98 (13.25)	72.38 (12.20)	−0.01	−0.59	1	0.556

Neurodevelopmental problem	n affected/total n	Any positive		*b*	z	OR	*P* value
		No (%)	Yes (%)				
ID	42/99	17 (36.2)	25 (48.1)	0.44	1.02	1.55	0.921
Any psychiatric disorder	53/108	33 (56.9)	20 (40)	0.76	1.87	2.13	0.062
ADHD	30/106	10 (20)	20 (35.7)	1.19	2.32	3.28	0.021
Any anxiety disorder	31/108	11 (22.0)	20 (34.5)	0.6	1.36	1.83	0.175
Any sleep problem	63/107	31 (62)	32 (56.1)	−0.24	−0.59	0.79	0.558
Indicative ASD	37/90	11 (25.6)	26 (55.3)	1.35	2.86	3.86	0.004
Indicative DCD	79/95	33 (73.3)	46 (92.0)	1.52	2.31	4.56	0.021

22q11.2DS, 22q11.2 deletion syndrome; SD, standard deviation; ADHD, attention‐deficit/hyperactivity disorder; ASD, autism spectrum disorder; DCD, developmental coordination disorder; FSIQ, full‐scale IQ, ID, intellectual disability; PIQ, performance IQ, VIQ, verbal IQ.

#### 3.3.2 Psychopathology, sleep disturbance, and motor coordination problems

We found higher rates of psychopathology, sleep disturbance, and indicative DCD in cases relative to controls (Table S1). Deletion carriers with ADHD were around three times more likely to have “any positive” and those with indicative ASD and DCD were around four times more likely (Table 5). The associations with indicative ASD and DCD held for “any positive excluding epilepsy” (*P* = 0.018 and *P* = 0.026, respectively), although the relationship with ADHD disappeared (*P* = 0.163). None of these relationships held for febrile seizures. None of the ESQ variables were predicted by psychiatric disorders as a group, anxiety disorder, or sleep disturbance. Age and gender did not influence our findings.

**Figure 1:**
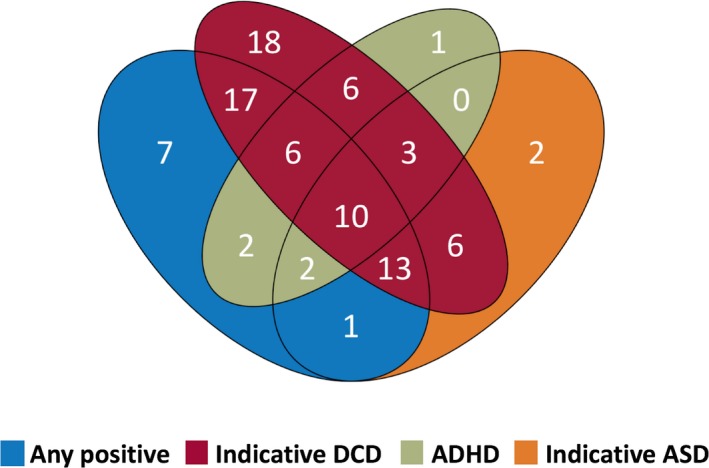
Overlap of “any positive” on the Epilepsy Screening Questionnaire, indicative DCD, ADHD, and indicative ASD in 22q11.2DS. DCD, developmental coordination disorder, ADHD, attention‐deficit/hyperactivity disorder, ASD, autism spectrum disorder; DCD, developmental coordination disorder

Overlap between “any positive” indicative DCD, ADHD, and indicative ASD was common in 22q11.2DS (Figure 1). Of those with indicative DCD, 58.2% (46/79) had “any positive,” as did 70.3% (26/37) with indicative ASD and 66.7% (20/30) with ADHD; 10.6% (10/94) screened positive in all four domains.

## 4 DISCUSSION

We showed that 11.1% of deletion carriers screened positive for an epilepsy diagnosis, a rate that falls within the wide‐ranging prevalence estimates from previous studies (4.4%‐36.8%^9^, ^13^, ^14^, ^15^, ^17^, ^18^). Over half of deletion carriers were, however, reported to have seizures or seizurelike symptoms without a diagnosis. In addition, 24.1% were reported to have febrile seizures. Results from a second stage of assessment (interview, prolonged ambulatory EEG, medical records, epileptologist review) indicated that rates of questionnaire‐reported epilepsy and febrile seizures were accurate and highlighted that in some deletion carriers, epileptic seizures may not be recognized during routine clinical care, and an epilepsy diagnosis may be overlooked. Deletion carriers who took part in the second assessment were older than those who did not, because this second assessment was conducted as part of a longitudinal study of development in young people with 22q11.2DS (Chawner et al^37^). Deletion carriers who screened positive on the epilepsy questionnaire had higher rates of ADHD, indicative ASD, indicative DCD, and a lower PIQ. These associations remained significant when cases with an epilepsy diagnosis were excluded, with the exception of ADHD, but none remained significant for febrile seizures. We propose that risk for epileptic seizures in deletion carriers may be symptomatic of underlying aberrant synaptic plasticity and an imbalance in neuronal excitation‐inhibition, also giving rise to impaired cognition, psychopathology, and motor coordination problems.

### 4.1 Epilepsy, seizures, and seizurelike symptoms in young people with 22q11.2DS

We observed an ~60% rate of ESQ‐reported seizures and seizurelike symptoms in deletion carriers, after excluding reported epilepsy cases. These events were more prevalent than in controls and showed many of the same associations with poorer neurodevelopmental outcomes as epilepsy. Our second assessment suggested that half of deletion carriers reported with these events may have had “true” epileptic seizures, mainly febrile and/or unprovoked. Crucially, this assessment also suggested that epileptic seizures may not be recognized during routine clinical care in some deletion carriers. We also newly diagnosed epilepsy in an individual with a clinical history of suspected seizures (nonmotor absences). Our findings are limited somewhat by the “possible” seizure attribution for two deletion carriers, due to remote epileptologist assessment. The ESQ may not detect some deletion carriers with clinically diagnosed epilepsy, as we found for one participant when cross‐referencing with medical records. Ultimately, prospective and systematic study of all ESQ‐reported seizures and seizurelike symptoms, incorporating direct epileptologist assessment, will best address the extent to which epileptic seizures and epilepsy are overlooked in 22q11.2DS.

#### 4.1.1 Febrile seizures

Our rate of reported febrile seizures in deletion carriers (24.1%) was higher than previous estimates (2%‐6%).^12^, ^13^, ^15^ This was not explained by the recurrent infections in this syndrome and suggests a reduced seizure threshold, supporting previous findings in adult deletion carriers with psychotropic and hypocalcemia‐induced seizures.^13^

Febrile seizures in deletion carriers resembled the “simple” phenotype, considered to be relatively benign.^38^ Supporting this, ESQ‐reported febrile seizures did not associate with poorer neurodevelopmental outcomes. Future longitudinal studies may better characterize the relationship of febrile seizures with neurodevelopmental trajectories in 22q11.2DS.

#### 4.1.2 Unprovoked seizures

The median age at onset of unprovoked seizures (33 months) and febrile seizures (14 months) suggests that deletion carriers are at risk for epileptic seizures from early in life. Unprovoked seizures were occurring once a day or more when at their most frequent in 30% of deletion carriers.

### 4.2 Association of the ESQ with neurodevelopmental problems in 22q11.2DS

The associations of ESQ‐reported epilepsy, seizures, and seizurelike symptoms with impaired cognition, psychopathology, and motor coordination problems in deletion carriers replicate findings from the general population^39^, ^40^, ^41^ and suggest shared neurobiologic risk pathways. One pathway could be aberrant synaptic plasticity and a subsequent imbalance in neuronal excitation‐inhibition. This mechanism associates with both epilepsy and autism.^42^ In a mouse model of ASD, aberrant synaptic plasticity was implicated in motor learning deficits via impaired cerebellar long‐term depression response and synaptic pruning.^43^ Synaptic plasticity in the prefrontal cortex is important in adolescent development of executive function, a behavioral impairment in ADHD.^44^ Impaired hippocampal synaptic plasticity shows links with memory deficits.^45^, ^46^ Aberrant synaptic plasticity is also implicated in the emergence of the sensory, cognitive, motor, and psychotic features that characterize schizophrenia,^47^ for which 22q11.2DS confers risk (22%).^7^ Mouse models of 22q11.2DS have demonstrated aberrant synaptic plasticity. For example, hemizygosity for the microRNA biogenesis gene *Dgcr8* leads to enhanced short‐ and long‐term synaptic plasticity within hippocampal CA3‐CA1 synapses, coinciding with spatial memory deficits, as well as enhanced short‐term depression in the prefrontal cortex. Mice that are haploinsufficient for the mitochondrial function gene *Mrpl40* also show abnormal short‐term potentiation within the hippocampus and co‐occurring working memory deficits. The aberrant synaptic plasticity in these mouse models is mediated by dysregulation of presynaptic calcium levels and neurotransmitter release (eg, enhanced glutamate activity).^45^, ^46^, ^48^

Epileptic seizures may also cause neurobiologic changes resulting in impaired cognition and ASD‐related behaviors, notably within the hippocampus^42^ and may alter cerebral functional organization of motor control.^49^ Paroxysmal epileptic activity associates with poorer performance on attention tests.^50^ Early life seizures may therefore have deleterious effects on vulnerable neural networks in 22q11.2DS, for example, within hippocampal and prefrontal regions,^18^, ^46^, ^48^ exacerbating impaired cognition, psychopathology, and motor coordination problems. Future longitudinal designs are warranted to assess this hypothesis.

### 4.3 Limitations of the Epilepsy Screen Questionnaire, and implications for our findings

The ESQ has high sensitivity for identifying epilepsy patients (96%) and a low false‐positive rate among seizure‐free individuals (7%). However, the estimated positive predictive value (PPV, the proportion of screen‐positive individuals who have epilepsy) in the general population is low (23%, possible range 0%‐100%^25^) reflecting the low prevalence of epilepsy (~1%^39^). The estimated rate of epilepsy in 22q11.2DS is higher (4.4%‐36.8%^9^, ^13^, ^14^, ^15^, ^17^, ^18^); however, false positives can be expected to occur as a consequence of the complex phenotypic presentation. The results of our second assessment do suggest, however, that most of the ESQ‐reported epilepsy diagnoses were genuine, and that half of deletion carriers reported with seizures or seizurelike symptoms may have had “true” epileptic seizures. Future studies replicating our method should ensure that all individuals with “any positive” take part in the second stage to establish the PPV of the ESQ for epileptic seizures in 22q11.2DS.

### 4.4 Clinical implications

Our work reinforces that not only are children with 22q11.2DS at risk of acute symptomatic seizures and epilepsy during the early years of life, but that in some, epileptic seizures may not be recognized during routine clinical care and an epilepsy diagnosis may be overlooked. We recommend a high‐index of suspicion for epileptic seizures in 22q11.2DS, with epileptologist consultation and video‐EEG for uncertain cases. Early intervention for epileptic seizures is important given the associations with poorer neurodevelopmental outcomes. A micro‐chromosomal array should be considered to screen for 22q11.2DS in children presenting with epileptic seizures and congenital heart disease, palatal abnormalities, hypocalcemia, ID, psychopathology, or motor coordination deficits.

In conclusion, we demonstrated that ~60% of young people with 22q11.2DS screen positive for seizures and seizurelike symptoms in the absence of an epilepsy diagnosis. Systematic investigation of a subsample showed that epileptic seizures may not be recognized during routine clinical care and that an epilepsy diagnosis may be overlooked. Risk for epileptic seizures in 22q11.2DS may be symptomatic of aberrant synaptic plasticity and an imbalance in neuronal excitation‐inhibition, also leading to cognitive impairment, psychopathology, and motor coordination problems. We observed a high rate of febrile seizures (24.1%), which provides further evidence for a reduced seizure threshold in 22q11.2DS. Highlighting the prevalence of epileptic seizures and epilepsy and their relationship with neurodevelopment in 22q11.2DS could allow focused intervention by clinicians, ultimately improving outcomes in this syndrome.

## DISCLOSURE OF CONFLICTS OF INTEREST

Ullrich Bartsch declares funding from Eli Lilly, and Rhys Thomas and Khalid Hamandi declare honoraria from Eisai, Sanofi, and UCB Pharma. The remaining authors have no conflicts of interest to disclose. We confirm that we have read the Journal's position on issues involved in ethical publication and affirm that this report is consistent with those guidelines.
