# Supplementary figures and images for: Epilepsy and seizures in young people with 22q11.2 deletion syndrome: Prevalence and links with other neurodevelopmental disorders
Source: Epilepsia. 2019 Apr 11;60(5):818–29. doi: 10.1111/epi.14722 (PMC6519005; doi:10.1111/epi.14722)

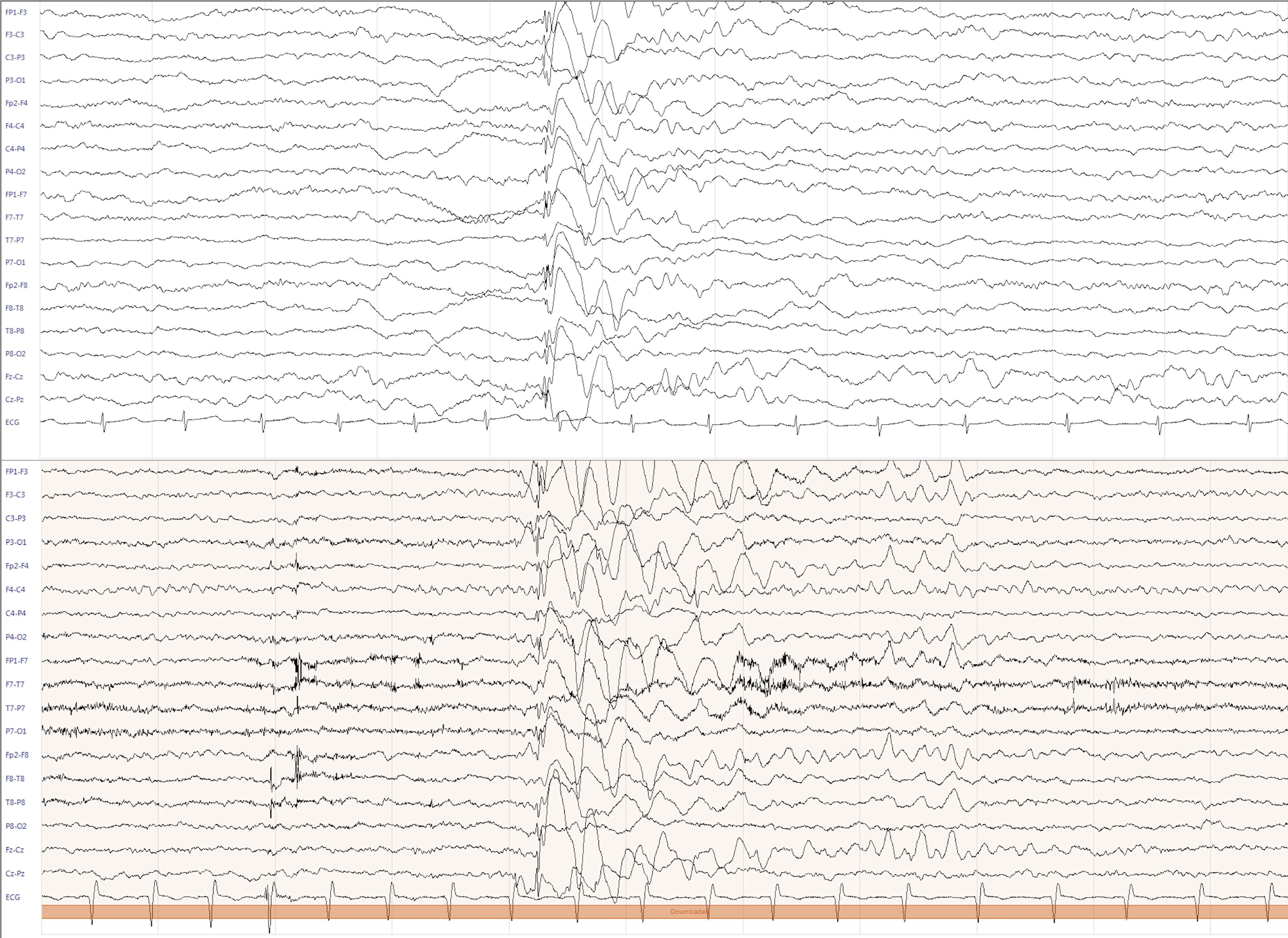

Supplement: Supplementary file 1 [file EPI-60-818-s001.tiff]
