# Supplementary material for: Epilepsy and seizures in young people with 22q11.2 deletion syndrome: Prevalence and links with other neurodevelopmental disorders
Source: Epilepsia. 2019 Apr 11;60(5):818–29. doi: 10.1111/epi.14722 (PMC6519005; doi:10.1111/epi.14722)
Supplement: Supplementary file 2 [file EPI-60-818-s002.pdf]

Supplementary Table 1. IQ scores and rates of neurodevelopmental problems in young people with 22q11.2DS and control siblings.

	22q11.2DS			Controls					
**Measure**	**n**	**Mean**	**s.d.**	**n**	**Mean**	**s.d.**	**t**	**95% CI**	**p-value**
**FSIQ**	99	72.05	12.29	55	108.58	15.3	15.19	31.76, 41.31	<0.001
**PIQ**	99	75.24	12.83	55	109.29	16.62	13.17	28.91, 39.18	<0.001
**VIQ**	100	73.13	12.67	55	106.45	14.77	14.12	28.64, 38.01	<0.001
**Neurodevelopmental problem**	**Total**	**Yes (%)**	**No (%)**	**Total**	**Yes (%)**	**No (%)**	χ^2	**OR**	**p-value**
**ID**	99	42 (42.4)	57 (57.6)	55	0(0)	55 (100)	32.08	-	<0.001
**Any psychiatric disorder**	108	53 (49.1)	55 (50.9)	53	2 (3.8)	51 (96.2)	32.44	24.19	<0.001
**ADHD**	106	30 (28.3)	76 (71.7)	47	1 (2.1)	46(97.9)	13.81	17.96	<0.001
**Any anxiety disorder**	108	31 (28.7)	77 (71.3)	49	2 (4.1)	47 (95.9)	12.3	9.36	<0.001
**Any sleep problem**	107	63 (58.9)	44 (41.1)	50	10 (20)	40 (80)	20.7	5.66	<0.001
**Indicative ASD**	90	37 (41.1)	53 (58.9)	48	0 (0)	48 (100)	26.96	-	<0.001
**Indicative DCD**	95	79 (83.2)	16 (16.8)	54	3 (5.6)	51 (94.4)	83.78	79.88	<0.001

FSIQ, full-scale IQ, PIQ, performance IQ, VIQ, verbal IQ, ID, intellectual disability, ADHD, attention deficit hyperactivity disorder, ASD, autism spectrum disorder, DCD, developmental coordination disorder.
